# Comparative effects of copper oxide nanoparticle morphology (nanorods vs. nanospheres) on the metabolism and swimming performance of *Oreochromis niloticus*

**DOI:** 10.1007/s10695-026-01821-x

**Published:** 2026-10-27

**Authors:** Edison Barbieri, Fabíola Sant’Ana Oliveira, Luiz Gustavo Ribeiro, Luelc Souza da Costa, Bruno Nunes

**Affiliations:** 1 Instituto de Pesca-APTA-Governo Do Estado de São Paulo, Cananeia, SP Brazil; 2 Development and Innovation Laboratory, Butantan Institute, São Paulo, SP Brazil; 3 Laboratório Nacional de Nanotecnologia (LNNano), Centro Nacional de Pesquisa Em Energia E Materiais (CNPEM), Campinas, SP Brazil; 4 Centro de Estudos Do Ambiente E Do Mar (CESAM), Universidade de Aveiro, Aveiro, Portugal; 5 Departamento de Biologia, Universidade de Aveiro, Aveiro, Portugal

**Keywords:** Nanotoxicology, Fish, Physiology, Ecotoxicology, Behavioral

## Abstract

The increasing production of nanomaterials has raised concerns regarding their environmental presence and potential adverse effects on non-target aquatic species. This study investigated the physiological impacts of acute (24 h) exposure of *Oreochromis niloticus* to copper oxide nanoparticles (CuO-NPs) of two distinct morphologies: nanorods (NRs) and nanospheres (NSs). Nanoparticles were synthesized and characterized via transmission electron microscopy, revealing significant differences in size, shape, and colloidal stability. Juvenile tilapia was exposed to concentrations ranging from 50 to 800 µg/L to evaluate routine metabolism (specific oxygen consumption), ammonia excretion, and swimming performance to fatigue. Both NRs and NSs significantly altered physiological parameters with distinct patterns. Oxygen consumption increased dose-dependently, reaching a 51% elevation with NRs (400 µg/L) and up to 254% with NSs (800 µg/L). Ammonia excretion was significantly inhibited in groups exposed to NRs. At 200 µg/L, excretion was nearly three times lower than in the control group, suggesting disturbances in gill function. In contrast, NSs induced significant increases in ammonia excretion (up to 127% at 800 µg/L), likely associated with enhanced protein catabolism. Swimming performance was also severely impaired, with NRs causing up to a 55% reduction in time to fatigue (200 µg/L), indicating locomotor and neuromuscular compromise. Comparative analysis showed that nanorods were consistently more toxic, supporting the “Trojan horse” hypothesis, likely due to their higher aggregation tendency and consequent release of CU^2+^ ions. These findings underscore the critical role of morphology, colloidal stability, and solubility in the ecotoxicological assessment of nanomaterials. We conclude that CuO-NPs, particularly in the form of nanorods, pose a significant risk to fish physiology and locomotor performance, reinforcing the need to integrate morphological, physiological, and behavioral endpoints in environmental risk assessment and regulatory policies for the safe use of these nanomaterials.

**Graphical abstract:**

**Figure:**
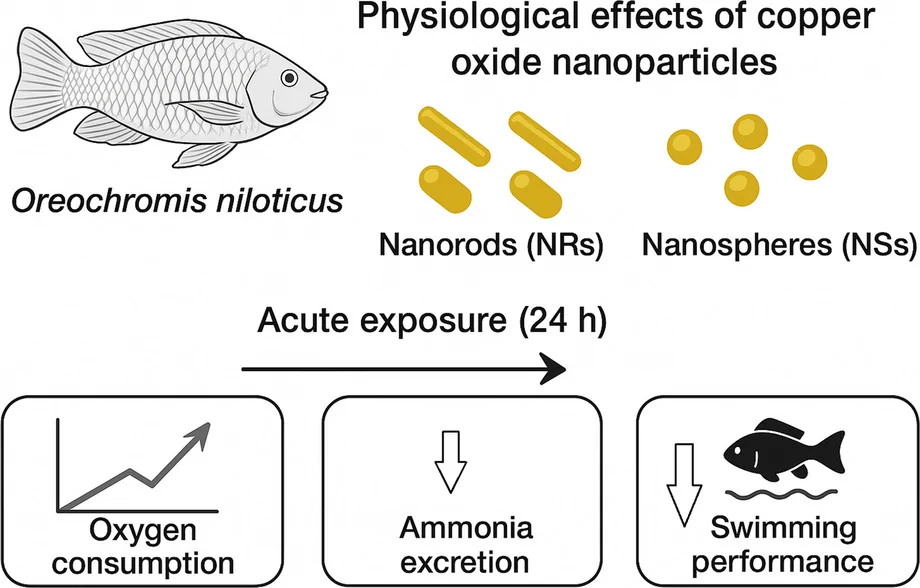

## Introduction

Nanotechnology involves the exploration and manipulation of particles within the 1–100-nm scale, where materials exhibit unique physicochemical properties due to an increased surface area-to-volume ratio (Alves et al. 2022). These modifications confer distinctive characteristics to nanoparticles compared to their bulk counterparts, even when sharing identical chemical composition (Midander et al. 2009). While these properties offer transformative possibilities across agri-food, pharmaceutical, and electronic sectors (Griffitt et al. 2008; Studer et al. 2010), the increasing use of nanomaterials has raised significant concerns regarding their environmental fate, particularly within aquatic ecosystems (Barbieri et al. 2019).

In aquatic systems, nanoparticles undergo physical and chemical transformations, such as aggregation and sedimentation, exposing resident organism to both dissolved ions and unique particulate forms (Griffitt et al. 2011; Aguiar et al. 2024; Dutta et al. 2025). Copper oxide nanoparticles (CuO-NPs), in particular, are widely incorporated into consumer products, ranging from food containers to antimicrobial coatings, due to their low production cost and diverse morphologies (Lu et al. 2017; Sielska and Skuza 2025; Keller et al. 2017; Liu et al. 2025). However, their widespread application facilitates potential release into the environment, where they may exert deleterious effects on non-target species (Wu et al. 2017).

The toxicity of CuO-NPs is largely associated with the release of Cu^+^ and Cu^2+^ ions, though the specific contribution of the particulate form remains a subject of debate (Song et al. 2015; Ghais et al. 2019; de Oliveira Eiras et al. 2022; Yahya 2023; Garncarek-Musiał et al. 2024). This uncertainty is central to the “Trojan horse” hypothesis, which suggests that nanoparticles act as stable vectors that facilitate the entry of metal into the organism. Once internalized or associated with biological membranes, these particles act as a reservoir, releasing high localized concentrations of toxic ions directly into cells or tissues, potentially causing greater damage than exposure to equivalent concentrations of dissolved metal alone (Assis da Silva et al. 2022).

Fish are particularly susceptible to these materials, with previous research identifying nanoparticles as drivers of morphological alterations in gills (Ostaszewska et al. 2016; Ribeiro et al. 2025), systemic metabolic shifts (de Oliveira Eiras et al. 2022), and behavioral impairments (Sovová et al. 2014). *Oreochromis niloticus* serves as a robust model for investigating these effects due to its global economic relevance and recognized sensitivity to environmental contaminants. Extensive literature has documented that Nile tilapia responds consistently to CuO-NPs through bioaccumulation (Abdel-Khalek et al. 2016; Tunçsoy et al. 2017), oxidative stress and cellular damage (Abdel-Latif et al. 2021; Yahya 2023), and disruptions in energy metabolism (Rezende et al. 2018; de Oliveira Eiras et al. 2022), qualifying the species as an efficient bioindicator for aquatic nanotoxicology (Ribeiro et al. 2025).

Despite these advances, substantial gaps persist in understanding how the physical shape of a nanoparticle modulates its physiological impact. We hypothesize that CuO-NP morphology (nanorods vs. nanospheres) significantly dictates toxicity levels by altering colloidal stability and the rate of ion release, thereby leading to differential impairments in fish energetics and swimming capacity.

Accordingly, this study aims to evaluate and compare the acute effects of CuO nanorods (NRs) and nanospheres (NSs) on the routine metabolism (oxygen consumption and ammonia excretion) and swimming performance to fatigue of juvenile *O. niloticus*. By integrating physiological and behavioral endpoints, we seek to determine if morphological differences in CuO-NPs result in distinct toxicological profiles, providing essential data for the environmental risk assessment of these nanomaterials.

## Materials and methods

### Synthesis of copper oxide nanoparticles

Copper oxide nanoparticles (CuO-NPs) with nanorod (NR) and nanosphere (NS) morphologies were supplied by the National Nanotechnology Laboratory (LNNano-CNPEM, Brazil). Both morphologies were synthesized using the alkaline coprecipitation method adapted from Misra et al. (2014).

#### Nanorods

Nanorods were synthesized from 0.02 M copper sulfate pentahydrate (CuSO_4_·5H_2_O) and glacial acetic acid at 100 °C, with the addition of 0.45 g NaOH under a N_2_ atmosphere. After 15 min, the system was cooled to room temperature, resulting in a precipitate containing CuO-NPs.

#### Nanospheres

Nanospheres were synthesized from 0.02 M copper sulfate pentahydrate (CuSO_4_·5H_2_O) and glacial acetic acid at 100 °C, with the addition of 0.90 g NaOH under a N_2_ atmosphere to modulate the nucleation and growth into spherical shapes.

For both types, the precipitate was purified by centrifugation at 5000 rpm (5590 g, using a rotor radius of 20 cm) for 5 to 10 min. To prevent aggregation and ensure colloidal stability, particles were resuspended in 1% (w/v) sodium citrate and sonicated for 20 min using a high-frequency sonicator before final storage in ultrapure water, protected from light.

#### Characterization

The morphological characterization was performed via transmission electron microscopy (TEM) using a JEOL JEM 2100 F microscope at 200 kV. Samples were sonicated for 20 min and deposited onto 400-mesh holey carbon grids to evaluate particle size, shape, and distribution.

### Test organism and acclimation

Juvenile Nile tilapia (*Oreochromis niloticus*) was obtained from the Fisheries Institute (APTA/SAA/SP, Cananéia, Brazil). All experimental procedures were conducted following the guidelines for animal welfare and were approved by the local Ethics Committee on Animal Use (CEUA).

A total of 88 fish (weight = 0.52 ± 0.12 g; length = 2.30 ± 0.25 cm) were acclimated for 1 week in 50-L aquaria. During this period, fish were kept under a natural photoperiod and fed daily with a commercial diet (24% crude protein) until 24 h prior to the assays, when feeding was suspended to establish fasting conditions.

### Experimental assays

The exposure concentrations for CuO-NPs (0, 50, 100, 200, 400, and 800 µg/L) were selected based on previous research evaluating the toxicological effects of metallic nanoparticles on aquatic organisms and preliminary range-finding experiments to ensure the assessment of dose-dependent sublethal effects.

Assays were conducted under static conditions in 5-L aquaria containing filtered tap water (0.45-µm membrane). Water quality parameters were maintained and monitored during the 24-h exposure period: temperature at 24 ± 1 °C, pH 6.64 ± 0.01, total hardness 18.66 ± 0.06 mg, CaCO_3_/L 6.64 (± 0.01), and conductivity 247.33 (± 16.46) µS/cm. Before addition to the aquaria, CuO-NP stock solutions were sonicated for 10 min to ensure proper dispersion. A negative control (untreated water) was used for all assays.

### Routine metabolism

After 24 h of exposure (nanorods and nanospheres) to concentrations of 0, 200, 400, and 800 µg/L (*n* = 6 per group), fish were transferred to individual 1000-mL cylindrical glass respirometers. Following a 1-h acclimation with continuous water flow to mitigate handling stress, the chambers were sealed for 1 h. After that, water samples were collected in amber borosilicate glass vials by siphoning. Dissolved oxygen concentrations were determined using the Winkler method (Nowaczyk Jr. et al. 1993), while ammonia excretion was measured using the Nessler method (Eaton et al. 1995). Oxygen consumption (mL O_2_/g/L/h) and ammonia excretion rates (mg/L/g/h) were calculated considering respirometer volume, fish wet weight, and confinement period (Damato and Barbieri 2012).

### Swimming performance

**Fig. 1:**
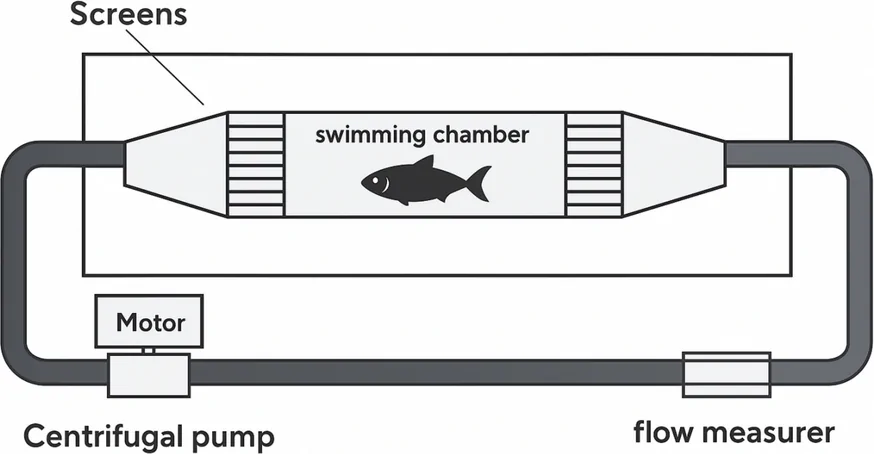
Schematic diagram of the 1.2-L hydrodynamic swim tunnel used for juvenile *O. niloticus* swimming performance assays at the Fisheries Institute (Cananéia-SP). The apparatus includes a swimming chamber, a centrifugal pump, and a flow meter system, adapted from Barbieri (2007)

Swimming capacity was assessed using a laboratory-built hydrodynamic tunnel (1.2 L), designed according to Brett (1964) and adapted for small fish (Barbieri 2007) (Fig. 1). Fish (*n* = 5 per group) were exposed to 0, 50, 100, and 200 µg/L of CuO-NPs for 24 h. After individual placement in the tunnel, water velocity was gradually increased until exhaustion, defined as the inability of the fish to maintain position against the flow for more than 60 s. Each fish was tested only once to avoid training bias.

### Statistical analysis

Data normality and homoscedasticity were verified using Shapiro–Wilk and Levene’s tests, respectively. Differences among groups were analyzed by one-way ANOVA, followed by Tukey’s post hoc test for multiple comparisons. All results are expressed as mean ± standard deviation (SD), with a significance level set at *p* < 0.05.

## Results

### Characterization of CuO-NPs (NRs and NSs)

**Fig. 2:**
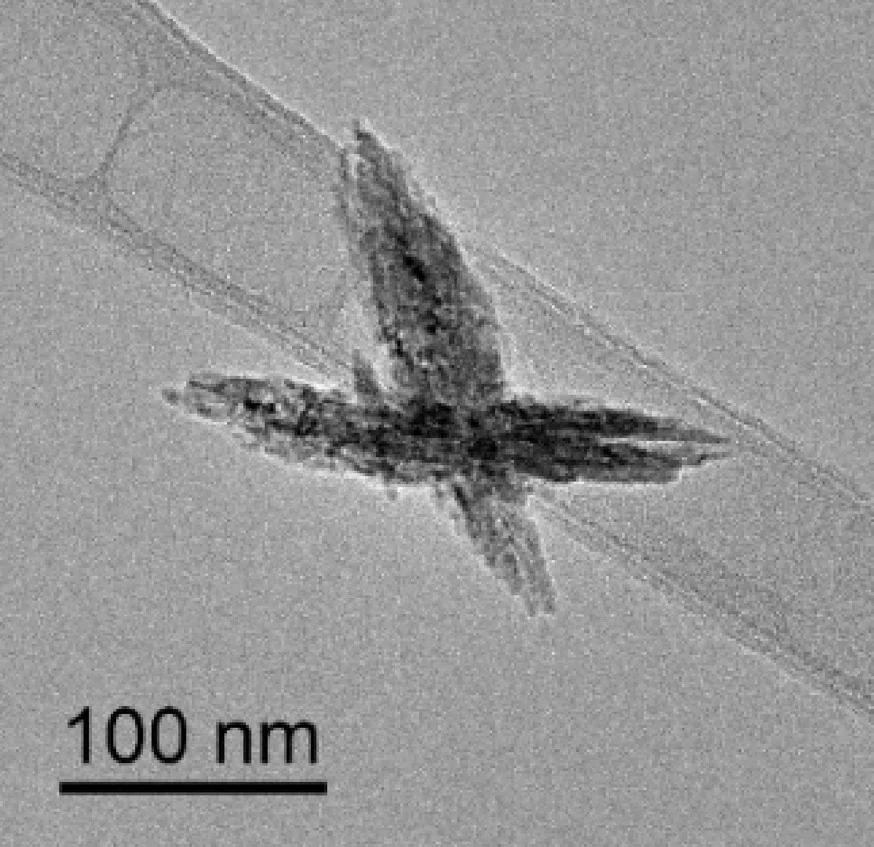
TEM images of CuO-NPs in nanorod morphology. Scale bar = 100 nm

Morphological characterization via transmission electron microscopy (TEM) confirmed the synthesis of two distinct copper oxide nanostructures: nanorods (NRs) and nanospheres (NSs). TEM images of the NRs revealed polycrystalline structures with multiple growth orientations and distinct crystallographic planes, indicating structural heterogeneity (Fig. 2).

**Fig. 3:**
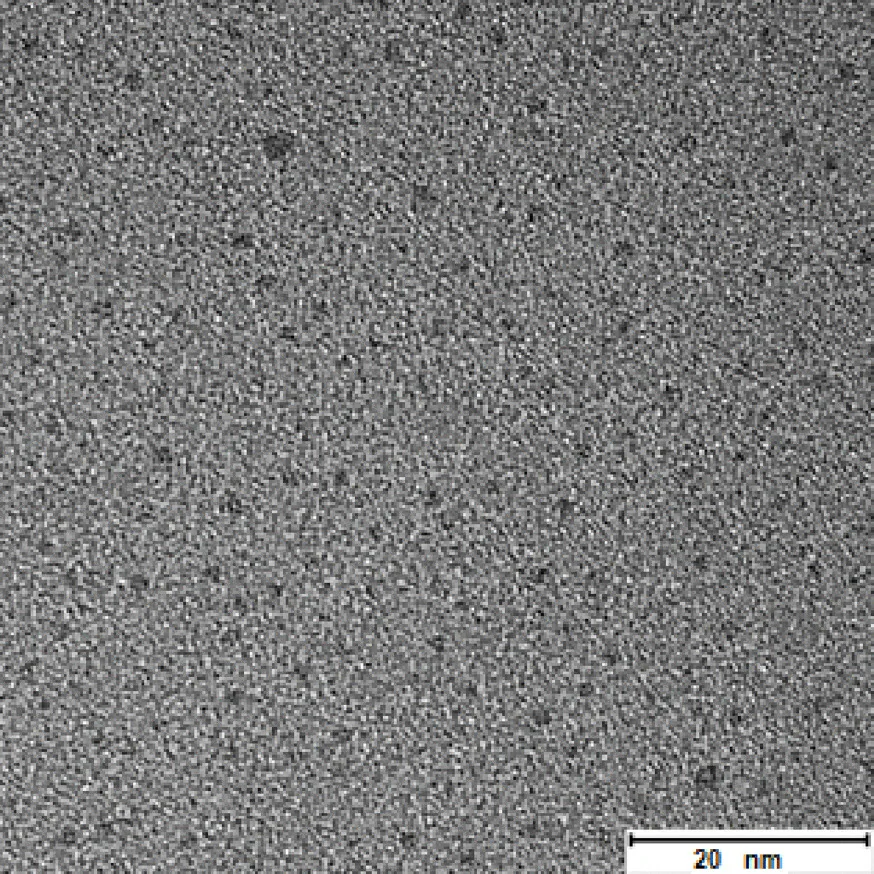
TEM images of CuO-NPs in nanosphere morphology. Scale bar = 30 nm

In comparison, nanorods exhibited significantly larger dimensions than nanospheres, suggesting a lower specific surface area. These physical features contribute to a higher aggregation tendency and rapid sedimentation in aquatic media, potentially affecting their bioavailability (Fig. 3).

In contrast, nanospheres displayed regular morphology with a uniform size distribution and no visible agglomeration. These properties indicate greater colloidal stability and a higher potential for homogeneous dispersion in biological systems, which may increase the frequency of particle–organism interactions.

### Specific oxygen consumption in fish exposed to CuO-NPs–nanorods

**Fig. 4:**
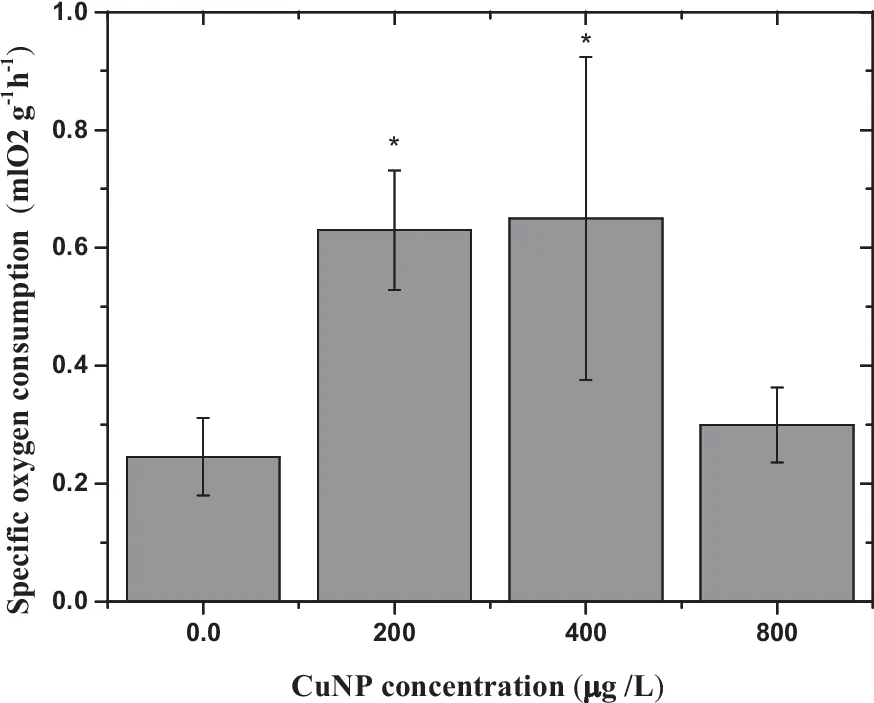
Specific oxygen consumption in fish exposed to different concentrations of CuO-NP nanorods. Values expressed as mean (*n* = 6) ± SD. *Statistically significant difference compared with control (*p* < 0.05)

Acute (24 h) exposure of fish to CuO-NPs in the nanorod form resulted in a significant increase in metabolic rate, measured as specific oxygen consumption (mL O_2_·g^−1^·h^−1^). At concentrations of 200 µg/L and 400 µg/L, oxygen consumption increased by 48% and 51%, respectively, compared to the control group (Fig. 4). These findings indicate higher energetic costs associated with homeostatic maintenance under nanoparticle-induced stress.

### Specific oxygen consumption in fish exposed to CuO-NPs–nanospheres

**Fig. 5:**
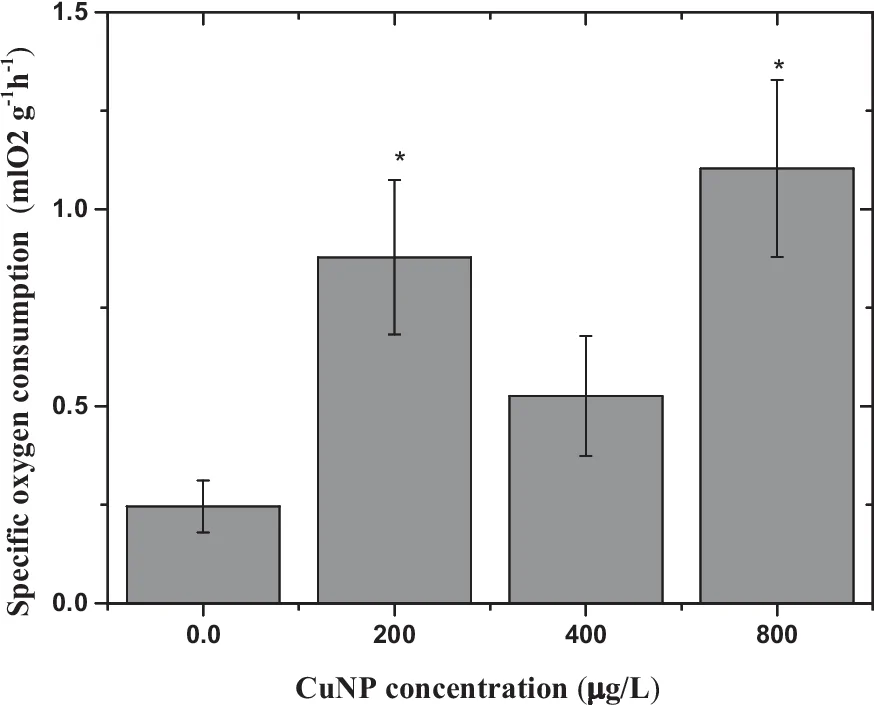
Specific oxygen consumption in fish exposed to different concentrations of CuO-NP nanospheres. Values expressed as mean (*n* = 6) ± SD. *Statistically significant difference compared with control (*p* < 0.05)

Exposure to CuO-NPs in nanosphere form elicited even more pronounced increases in metabolic rate. At 200 µg/L and 800 µg/L, oxygen consumption increased by 171% and 254%, respectively, compared with controls (Fig. 5). These results suggest that, despite their morphological stability, NSs can trigger intense physiological effects, possibly due to higher bioavailability and enhanced cellular uptake.

### Ammonia excretion in fish exposed to CuO-NPs–nanorods

**Fig. 6:**
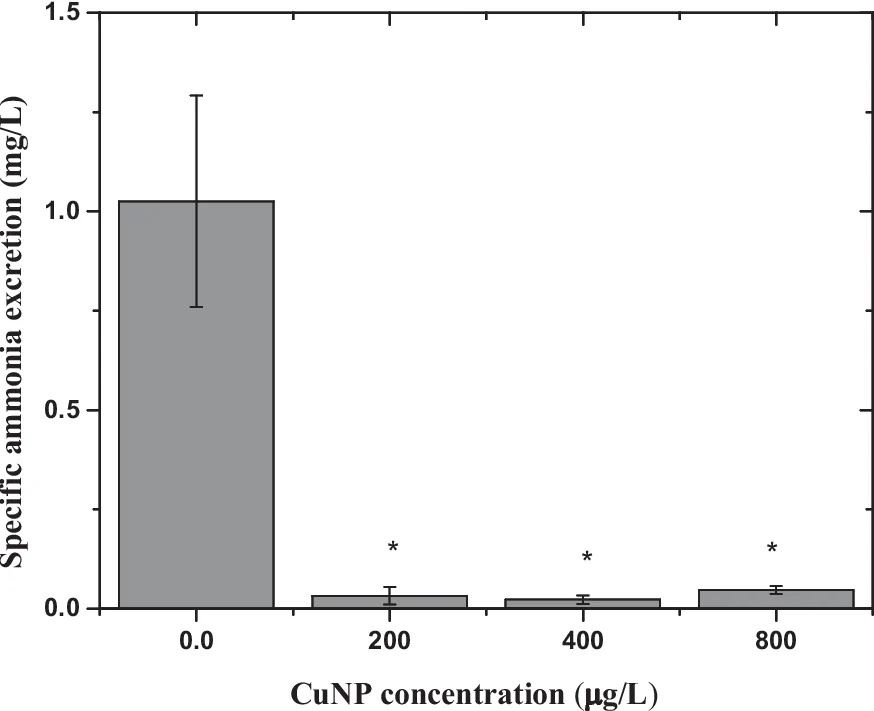
Specific ammonia excretion in fish exposed to different concentrations of CuO-NP nanorods. Values expressed as mean (*n* = 6) ± SD. *Statistically significant difference compared with control (*p* < 0.05)

Ammonia excretion was significantly reduced in fish exposed to CuO-NP nanorods at 200 µg/L and 800 µg/L, with reductions of 187% and 93%, respectively, compared with the control group (Fig. 6). This reduction may reflect impairment of excretory function or interference with nitrogen detoxification pathways, suggesting possible renal or hepatic toxicity.

### Ammonia excretion in fish exposed to CuO-NPs–nanospheres

**Fig. 7:**
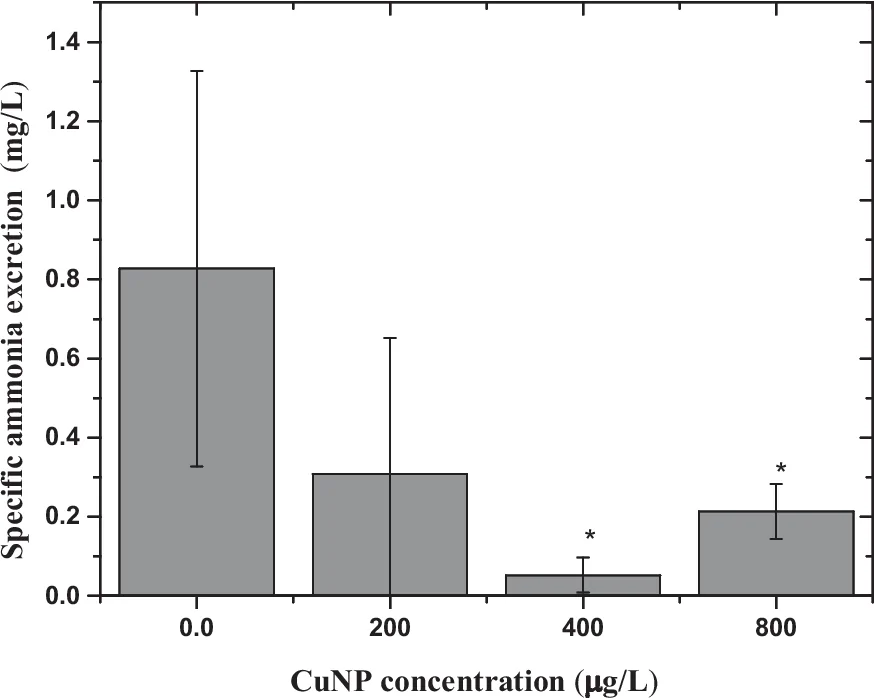
Specific ammonia excretion in fish exposed to different concentrations of CuO-NP nanospheres. Values expressed as mean (*n* = 6) ± SD. *Statistically significant difference compared with control (*p* < 0.05)

In contrast to nanorods, nanospheres induced a significant increase in ammonia excretion at 400 µg/L and 800 µg/L, with increments of 125% and 127%, respectively (Fig. 7). This response may represent a compensatory mechanism linked to enhanced protein catabolism, consistent with the elevated metabolic rates observed.

### Swimming performance after exposure to CuO-NP nanospheres

**Fig. 8:**
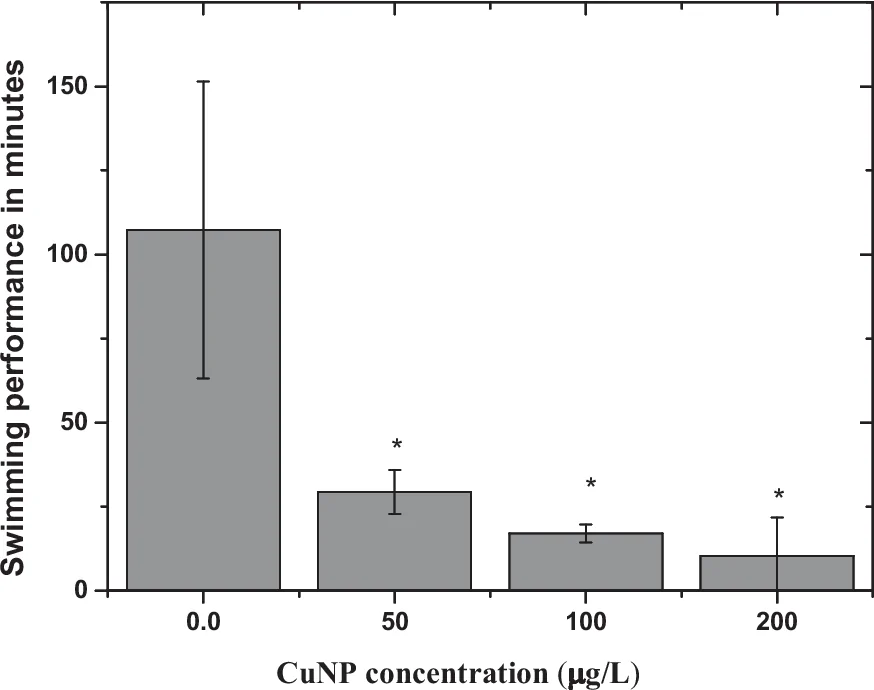
Swimming performance of fish exposed to different concentrations of CuO-NP nanospheres. Values expressed as mean (*n* = 5) ± SD. *Statistically significant difference compared with control (*p* < 0.05)

Swimming performance analysis demonstrated that fish exposed to CuO-NP nanospheres exhibited a significant decline in locomotor capacity with increasing concentrations (Fig. 8). Reduced endurance may indicate neurophysiological impairment or muscular dysfunction associated with nanoparticle toxicity.

### Swimming performance after exposure to CuO-NP nanorods

**Fig. 9:**
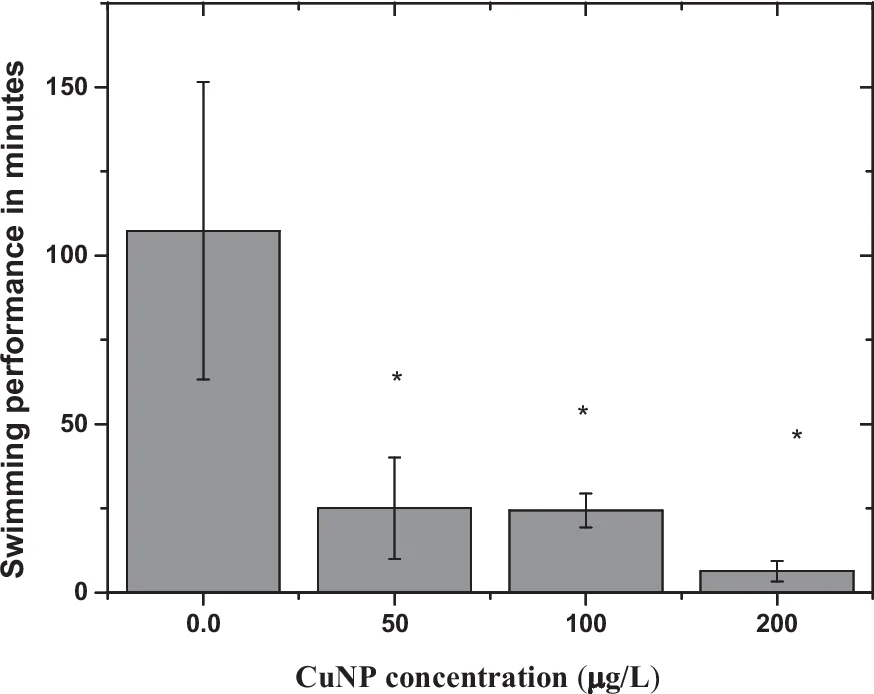
Swimming performance of fish exposed to different concentrations of CuO-NP nanorods. Values expressed as mean (*n* = 5) ± SD. *Statistically significant difference compared with control (*p* < 0.05)

Similarly, nanorods negatively affected swimming capacity, with marked reductions in physical endurance at the tested concentrations (Fig. 9). This response may be related to oxidative stress, mitochondrial dysfunction, or systemic physiological alterations, as indicated by other parameters measured.

## Discussion

The exposure of *Oreochromis niloticus* to copper oxide nanoparticles (CuO-NPs), in both nanorod (NR) and nanosphere (NS) forms, induced significant alterations in routine metabolism, ammonia excretion, and swimming performance. These responses differed according to concentration and nanoparticle morphology, supporting the relevance of physicochemical features in the ecotoxicological assessment of metal-based nanomaterials in aquatic species.

Specific oxygen consumption increased significantly in fish exposed to CuO-NP NRs, with elevations of 48% and 51% at 200 and 400 µg/L, respectively. This pattern suggests an increased energetic demand associated with the maintenance of physiological homeostasis under nanoparticle exposure. Changes in oxygen consumption are commonly interpreted as indicators of metabolic adjustment in fish exposed to environmental contaminants, since higher oxygen uptake may reflect increased energetic costs related to detoxification, ionoregulation, repair mechanisms, or stress responses. However, because molecular and cellular biomarkers were not assessed in the present study, these mechanisms should be interpreted as plausible explanations rather than directly demonstrated pathways.

The toxic effects of CuO-NPs may be influenced by both particle-specific properties and the release of dissolved copper ions. Previous studies have shown that CuO-NPs can undergo partial dissolution, releasing Cu^2+^ ions that may interfere with ionic homeostasis and cellular metabolism (Song et al. 2015; Naz et al. 2020; Yahya 2023). The “Trojan horse” hypothesis has also been proposed to explain nanoparticle toxicity, in which particles are internalized and subsequently release metal ions within biological compartments. Nevertheless, the relative contribution of particulate CuO-NPs and dissolved Cu^2+^ remains difficult to determine without direct measurements of dissolution kinetics in the exposure medium or biological tissues. Therefore, although Cu^2+^ release may have contributed to the effects observed here, this interpretation remains a limitation of the study.

In this context, Black et al. (2017) demonstrated that copper-based nanoparticles may alter oxygen consumption even when dissolved copper remains undetectable, indicating that particle-specific interactions with biological surfaces can also contribute to toxicity. Similarly, studies with other fish species have shown that copper and nano-copper exposure may affect metabolic regulation, oxidative balance, and stress-related responses (Malhotra et al. 2020; Dutta et al. 2025). These findings reinforce that the physiological effects of copper-based nanoparticles are complex and may result from the combined influence of particle morphology, aggregation behavior, bioavailability, and ion release.

Ammonia excretion was significantly reduced in fish exposed to CuO-NP NRs, particularly at 200 and 800 µg/L. Because ammonia elimination in freshwater fish occurs predominantly through the gills, this response may indicate an impairment of nitrogen excretion or gill-associated physiological processes. Previous studies have shown that copper exposure can interfere with ionoregulatory and ammonia transport pathways in fish, including processes involving ionocytes and Rh glycoproteins (Lim et al. 2015; Lee et al. 2020). However, since gill histology, ionocyte morphology, Rh glycoprotein expression, and renal or hepatic biomarkers were not measured in the present study, it is not possible to identify the specific biological target responsible for the observed reduction in ammonia excretion.

In contrast to NRs, CuO-NP NSs induced significant increases in ammonia excretion at higher concentrations. This response may be associated with enhanced protein catabolism or altered nitrogen metabolism under increased energetic demand, as suggested by the concomitant increase in oxygen consumption. Nevertheless, this interpretation should be considered with caution, since enzymatic pathways, protein metabolism, and nitrogen-related molecular markers were not directly evaluated. Therefore, the divergent responses observed between NRs and NSs suggest that nanoparticle morphology may influence metabolic and excretory outcomes, but additional biochemical and molecular endpoints are required to clarify the mechanisms involved.

Swimming performance was also impaired after CuO-NP exposure, with NRs causing reductions of up to 55% in time to fatigue. Swimming performance is an integrative endpoint that depends on respiratory capacity, cardiovascular function, muscle metabolism, neuromuscular coordination, and overall energetic status. Therefore, the reduction observed in exposed fish may reflect a broader physiological impairment rather than a single toxic mechanism. Previous studies have reported that copper exposure can affect fish behavior, including locomotor activity, avoidance responses, foraging behavior, and chemosensory function (Malhotra et al. 2020). In this sense, the reduction in swimming performance observed in the present study is ecologically relevant, as impaired locomotor capacity may compromise predator avoidance, prey capture, dispersal, and survival.

The stronger and more consistent effects observed in fish exposed to CuO-NP NRs suggest that nanoparticle morphology is an important factor influencing toxicity. Differences in shape, surface reactivity, aggregation behavior, sedimentation, and dissolution may alter the interaction of nanoparticles with fish gills and other biological surfaces. Kennedy et al. (2013) showed that CuO nanomaterials with different morphologies can differ in fate and toxicity, supporting the need to consider morphology in nanomaterial risk assessment. However, because hydrodynamic size, zeta potential, aggregation behavior, and dissolved copper concentrations were not measured in the exposure medium in the present study, the mechanisms underlying the higher toxicity of NRs cannot be conclusively determined.

This study has important limitations that should be acknowledged. First, nanoparticle characterization was based on TEM morphology, but no measurements of hydrodynamic diameter, polydispersity index, zeta potential, aggregation behavior, or copper dissolution in the exposure medium were available. These parameters are particularly important for interpreting nanoparticle behavior in aquatic toxicity assays. Second, the absence of a dissolved copper positive control limits the ability to distinguish nanoparticle-specific effects from ion-mediated toxicity. Third, oxidative stress biomarkers, gene expression, histopathology, and enzymatic assays were not assessed, limiting mechanistic interpretation. Despite these limitations, the integration of metabolic, excretory, and behavioral endpoints provides evidence that CuO-NPs can impair physiologically relevant functions in *O. niloticus*, with morphology-associated differences in toxicity.

Overall, the findings indicate that CuO-NPs affect energy metabolism, nitrogen excretion, and swimming performance in juvenile Nile tilapia. The results also suggest that nanorods may induce stronger physiological disturbances than nanospheres under the tested conditions. These responses reinforce the importance of incorporating nanoparticle morphology and organism-level endpoints into ecotoxicological evaluations of engineered nanomaterials. Future studies should combine physiological endpoints with nanoparticle stability analysis, dissolved metal quantification, oxidative stress biomarkers, gene expression, and histological assessment to better define the mechanisms underlying CuO-NP toxicity in fish.

## Conclusion

This study shows that copper oxide nanoparticles (CuO-NPs) can induce sublethal physiological and behavioral alterations in juvenile *Oreochromis niloticus*, with responses varying according to concentration and nanoparticle morphology. Under the tested conditions, CuO nanorods (NRs) produced stronger and more consistent effects than nanospheres (NSs), particularly through reductions in ammonia excretion and swimming performance. Both morphotypes also increased oxygen consumption, indicating higher energetic demand in exposed fish. The distinct responses observed for ammonia excretion suggest that NRs and NSs may interfere differently with metabolic and excretory processes. However, because gill morphology, oxidative stress biomarkers, gene expression, copper dissolution, and nanoparticle stability in the exposure medium were not assessed, the mechanisms underlying these effects cannot be conclusively determined. Therefore, interpretations related to gill impairment, ion-mediated toxicity, or altered protein catabolism should be considered plausible hypotheses requiring further investigation.

Overall, the integration of routine metabolism, ammonia excretion, and swimming performance proved useful for detecting sublethal effects of CuO-NPs in *O. niloticus*. These findings reinforce the importance of considering nanoparticle morphology in ecotoxicological assessments and highlight the need for future studies combining organism-level endpoints with physicochemical characterization in exposure media, including hydrodynamic size, aggregation behavior, zeta potential, and copper dissolution. Such information is essential to improve environmental risk assessment of CuO-NPs and to support safer production, use, and disposal of metallic nanomaterials.

## Data Availability

The datasets generated and/or analyzed during the current study are available from the corresponding author on reasonable request.
